# Comparative analysis of phytochemical compounds and quality characteristics during the juvenile stage of hazelnut cultivars from Italy and Türkiye

**DOI:** 10.1186/s12870-025-07673-6

**Published:** 2025-11-19

**Authors:** Burhan Ozturk, Huseyin Noyan, Serkan Uzun, Selim Karagol, Halil Erdem, Mert Ondin

**Affiliations:** 1 Department of Horticulture, Faculty of Agriculture, Ordu University, Ordu, Türkiye; 2 Department of Plant and Animal Production, Çilimli Vocational School, Düzce University, Düzce, Türkiye; 3 Department of Horticulture, Faculty of Agriculture, Tokat Gaziosmanpaşa University, Tokat, Türkiye; 4 Department of Horticulture, Faculty of Agriculture, Ondokuz Mayıs University, Samsun, Türkiye

**Keywords:** Antioxidant activity, *Corylus Avellana* L., Fatty acid composition, Linoleic acid, Phenolics, Kernel characteristics

## Abstract

**Background:**

The present study aimed to comparatively analyze phytochemical compounds and quality characteristics of hazelnut cultivars from Italy (‘Tonda Gentile Romana’, ‘Tonda di Giffoni’, ‘Nocchione’, and ‘Mortarella’) and Türkiye (‘Tombul’, ‘Foşa’, and ‘Çakıldak’), cultivated under identical ecological conditions. In this scope, a comprehensive evaluation of the physical characteristics of the nuts and kernels, biochemical properties (total phenolics, total flavonoids, antioxidant activity), protein, oil, ash contents, and fatty acid composition were conducted in cultivars.

**Results:**

A wide variation was observed in the characteristics investigated among hazelnut cultivars. In the study, physical characteristics were determined as nut weight between 1.82 and 3.31 g, kernel weight between 0.96 and 1.49 g, shell thickness between 1.10 and 1.96 mm, and kernel percentage between 39.61 and 52.87%. The biochemical properties included total phenolics, total flavonoids, and antioxidant activity (according to DPPH and FRAP assays), which ranged from 21.84 to 50.86 g GAE kg^− 1^, 3.11 to 6.09 g QE kg^− 1^, 13.15 to 37.51 mmol TE kg^− 1^, and 57.22 to 102.13 mmol TE kg^− 1^, respectively. The oil content was found to vary from 52.46 to 65.74%. Also, palmitic acid (C_16:0_) varied between 4.66 and 5.79%, stearic acid (C_18:0_) varied between 1.71 and 2.76%, oleic acid (C_18:1_) varied between 83.21 and 87.37%, and linoleic acid (C_18:2_) varied between 4.25 and 8.77%. Principal component analysis indicated that the examined characteristics were highly effective in describing the variation among cultivars, with the first three principal components accounting for 84.9% of the overall variability.

**Conclusions:**

This comparative study demonstrates considerable variation in physical and biochemical characteristics among Italian and Turkish hazelnut cultivars. Results provide important information for future cultivar selection and breeding programs, highlighting characteristics desirable for specific commercial and industrial treatments.

## Introduction

Hazelnut (*Corylus avellana L.*) is a widely cultivated nut known for its extensive usage and significant economic importance [1, 2]. Approximately 70% of the world’s hazelnut production is used in the chocolate industry, 20% in ice cream and pastry, while only 10% is consumed directly [3]. Türkiye and Italy are the leading countries in terms of production area, cultivar richness, and production amounts. Based on 2023 data from the Food and Agriculture Organization of the United Nations, Türkiye produced 650.000 t of hazelnuts across an area of 746.749 ha, whereas Italy’s production was 102.740 t from 87.500 ha [4]. Although traditionally both countries have a long history of hazelnut cultivation, they have significant differences in terms of cultivar differences, climatic conditions, and orchard management [5].

Türkiye is the country that produces and exports the most hazelnuts in the world [6]. The Black Sea region in the north of the country has ideal agroclimatic conditions, very suitable for hazelnut cultivation [7]. Cultivars such as ‘Tombul’, ‘Palaz’, ‘Çakıldak’, ‘Foşa’, and ‘Mincane’, which have been well adapted to the region’s humid climate for many years, are widely grown and have a high commercial value [8]. On the other hand, cultivars originating from Italy are known for their high-quality kernels and varieties suitable for industrial processing. In Italy, 98% of hazelnut cultivation takes place in the provinces of Campania, Lazio, Piemonte, and Sicilia, and the main cultivars produced are ‘Tonda Gentile delle Langhe’, ‘Tonda Gentile Romana’, ‘Tonda di Giffoni’, ‘San Giovanni’, ‘Mortarella’, and ‘Riccia di Talanico’ [9].

Hazelnuts, which have come to the forefront for many years with their rich nutritional content including lipids, dietary fiber, protein, minerals, and vitamins [10], can be consumed fresh or roasted and are widely used in the production of products such as chocolate, pastry, ice cream, and cake [11]. Additionally, hazelnuts contain proteins, fatty acids, and phenolic compounds that can inhibit or slow down lipid oxidation and neutralize free radicals [12]. These bioactive components enhance antioxidant activity, potentially lowering the risk of chronic diseases, including cardiovascular diseases, diabetes, and cancer [13]. Studies on hazelnut cultivars cultivated within the same geographical area demonstrate substantial differences in their phytochemical composition and nutritional quality. Even when grown under identical conditions, distinct cultivars can show notable variation in protein, fat, fiber, mineral, and tocopherol contents [14]. The main factors that influence the quality of hazelnuts and their bioactive content are genetic structure, ecology, climate, fertilization, pruning, irrigation, harvest time, and diseases and pests [12, 15].

Although previous studies have examined the physical and biochemical quality characteristics of hazelnut cultivars, research comparing cultivars grown under identical ecological conditions remains limited. Therefore, the main aim of this study was to make a comprehensive comparison of seven hazelnut cultivars, four Italian (‘Tonda Gentile Romana’, ‘Tonda di Giffoni’, ‘Nocchione’, and ‘Mortarella’) and three Turkish (‘Tombul’, ‘Foşa’, and ‘Çakıldak’), grown under the same ecological conditions. Detailed quality traits of Turkish and Italian hazelnut varieties will be examined under the same ecological conditions, addressing this research gap. Within the scope of the study, physical nut and kernel characteristics, protein, oil, and ash contents, biochemical characteristics, and fatty acid composition of the cultivars were determined and compared.

## Materials and methods

### Plant material

The plant material of the study consisted of ‘Tonda Gentile Romana’, ‘Tonda di Giffoni’, ‘Nocchione’, and ‘Mortarella’ cultivars in Ordu University, Faculty of Agriculture, Application and Research Orchard. In addition, the cultivars ‘Tombul’, ‘Foşa’, and ‘Çakıldak’, which were grown under similar conditions, were used as positive controls. Tissue-cultured seedlings were obtained from a private nursery (Vivai Vignolini company) in Viterbo (Italy), and subsequently planted in the field in March 2018, with a distance of 5.0 × 4.0 m. The plants propagated from the sucker of these plants were cultivated as potted seedlings in 2019 and then planted in the experimental plot in 2020. The soil structure of the experimental area is clay-loamy with medium organic matter content. Cultural practices such as irrigation, fertilization, weed control, disease and pest control were carried out regularly. Irrigation was provided according to plant water needs by the drip irrigation method with a double-line drip irrigation system with 2 L/h drippers. A total of 100 g nitrogen (N) per plant was applied in two equal portions during March and May, while 15 g of monoammonium phosphate (NH_4_H_2_PO_4_) and 30 g of potassium sulfate (K_2_SO_4_) were applied in October.

The experiment was designed with three replicates, with three plants of each cultivar used in each replicate. The nuts that reached harvest maturity in each plant were manually collected and dried in the sun.

The harvest times for the cultivars are documented in the following: for ‘Tombul’ the first week of August; for ‘Foşa’, and ‘Çakıldak’, the last week of August; and for ‘Tonda Gentile Romana’, ‘Tonda di Giffoni’, ‘Nocchione’, and ‘Mortarella’ the last week of September. For each repetition, 100 dried fruits selected at random were used for measurement and analysis. According to the climate data for many years in Ordu (1959–2024), the mean highest temperature is 18.5 °C, the lowest temperature is 11.3 °C, the annual average temperature is 14.6 °C, and the annual average precipitation is 1049.8 mm [16]. The measurements and analyses were conducted in 2022 and 2023, with the mean values for these two years presented in the study.

### Nut characteristics

Nut weight (Nwe) (g) and kernel weight (Kwe) (g) were determined by using a digital scale (Radwag, Poland) with a precision of 0.01 g. Shell thickness (ST), nut and kernel dimensional characteristics (width, length, thickness) were measured using a digital caliper (Mitutuyo, Japan) with a precision of 0.01 mm. The kernel percentage (KP) (%) was determined by calculating the ratio of the total nut weight to the total good kernel weight [KP (%) = (Kwe/Nwe) x 100] [17]. The number of nuts in 100 g (NN100) (pcs) and the number of kernels in 100 g (NK100) (pcs) were determined by counting the number of nuts and kernels weighed in 100 g for each cultivar. The weight of 100 nuts (WN100) (g) and the weight of 100 kernels (WK100) (g) were determined with a digital scale.

### Crude protein (%) and Ash (%)

Crude protein content (PC) and ash content (AC) were determined using standard procedures outlined by the Association of Official Analytical Chemists [18]. The PC was determined by using the *Kjeldahl* method, and the result was then calculated by multiplying the value of nitrogen (N) by a conversion factor of 6.25 [PC (%) = N x 6.25]. Ash analysis was conducted in accordance with the dry ashing method at 550 °C. Following the incineration of the samples in a furnace, the resulting ash was weighed using a digital scale [17].

### Crude oil content (%) and fatty acid composition (%)

**Fig. 1:**
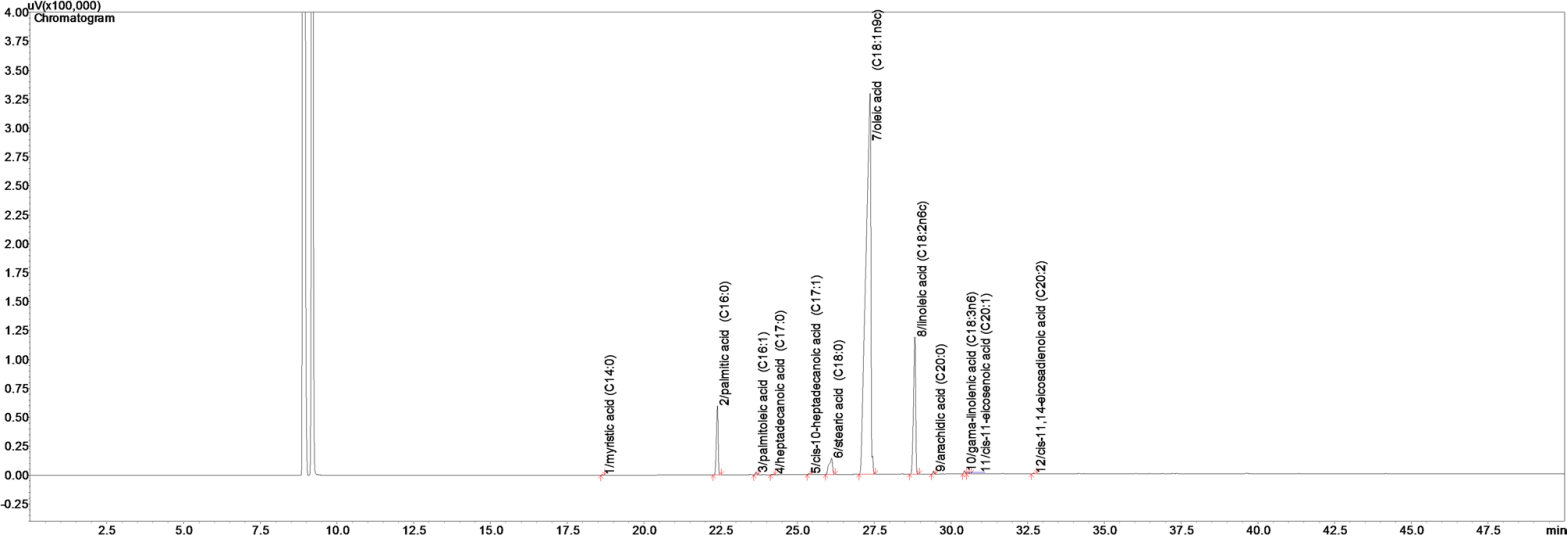
GC-FID chromatogram of fatty acids

Crude oil content (OC) was determined by the Soxhlet extraction method [19]. 5 g hazelnut kernel powder was weighed into Soxhlet cartridges. Then samples were treated with n-hexane for four hours at 130 °C using Soxhlet extraction apparatuses. Results were expressed as a percentage of dry matter. The fatty acid composition of oils obtained from hazelnut kernels was determined by gas chromatography (Shimadzu, Kyoto, Japan) (GC) according to the method reported by Karakaya [12]. The method for GC-FID analysis has been described in more detail in manuscript. 0.1 g of hazelnut oil was placed into a test tube, followed by the addition of 1 mL of potassium methylate and 4 mL of hexane. The mixture was vigorously shaken for 30 s, after which 0.5 mL of 25% sulfuric acid (H₂SO₄) was added. The resulting solution was then diluted with hexane and filtered through a 0.45 μm membrane filter. Subsequently, the prepared fatty acid methyl esters (FAMEs) were analyzed using a GC (Shimadzu, Kyoto, Japan) equipped with a flame ionization detector (FID). Separation was carried out on a capillary column (TR-CN100, Teknokroma, Barcelona, Spain) with dimensions of 0.25 mm × 0.20 μm and 100 m in length. The column temperature program began at 140 °C (held for 5 min), increased at a rate of 4 °C/min to 240 °C, and was maintained at that temperature for 15 min. The injector and detector temperatures were both set at 250 °C, and nitrogen was used as the carrier gas at a flow rate of 3 mL/min, in accordance with the method. FAMEs were identified by comparing their retention times with those of commercial standards, and results were expressed as the percentage of each fatty acid based on relative peak area (Fig. 1).

### Biochemical characteristics

The total phenolic content (TPC), total flavonoids content (TFC), and antioxidant activity (AA) [according to 1,1-diphenyl-2-picrylhydrazyl (DPPH) and ferric reducing antioxidant power (FRAP) assays] were measured by using a spectrophotometer (Shimadzu). TPC was determined by the Folin-Ciocalteu reagent, following the method described in the report by Karakaya et al. [20]. 1000 µL of the stock solution prepared for the total phenolic content determination was taken and 3600 µL of distilled water, 100 µL of Folin-Ciocalteu reagent, and 300 µL of sodium carbonate (Na₂CO₃) were added. The prepared solution was incubated for 2 h. After incubation, the samples were read at a wavelength of 760 nm using a spectrophotometer. The values obtained were calculated in terms of gallic acid and expressed as g GAE kg^− 1^.

The TFC was determined in accordance with the method detailed in the report by Chang et al. [21]. The TFC was determined in accordance with the method detailed in the report by Chang et al. [20]. 1000 µL of the prepared stock solution was taken, and 3300 µL of methanol, 100 µL of aluminum nitrate [Al(NO₃)₃], and 100 µL of ammonium acetate (NH₄CH₃COO) were added to it. The prepared solution was incubated for 30 min. After incubation, the solution was read at 415 nm in a spectrophotometer, and the obtained values were expressed as quercetin content in g QE kg^− 1^.

Measurements of AA were conducted using the FRAP assay reported by Benzie and Strain [22], and the DPPH assay reported by Blois [23]. To determine antioxidant activity using the FRAP method, 20 µL was taken from the prepared stock solution, and 1230 µL of phosphate buffer and 1250 µL of potassium ferricyanide were added. The prepared solution was incubated in a water bath at 50 °C for 25 min. After incubation, 1250 µL of TCA and 250 µL of ferric chloride were added to the samples. The prepared sample was read at a wavelength of 700 nm in a spectrophotometer, and the obtained values were expressed as mmol TE kg^− 1^. DPPH assay; 30 µL was taken from the prepared stock solution, and 2970 µL of ethyl alcohol and 0.26 mM 1000 µL DPPH solution were added to it. The prepared solution was left to incubate for 30 min. After incubation, the samples were read at 517 nm wavelength using a spectrophotometer, and the obtained values were expressed as mmol TE kg^− 1^.

### Statistical analysis

To assess the normality of the data distribution, the Kolmogorov-Smirnov test was applied. Homogeneity of variances among groups was evaluated using Levene’s test. Following the analysis of variance (ANOVA), Tukey’s post-hoc test was conducted to identify statistically significant differences among the groups at the 5% significance level (*p* ≤ 0.05). All statistical procedures were performed using SAS software (version 9.1, USA). Additionally, principal component analysis (PCA), biplot, and heatmap visualizations based on the physical and biochemical characteristics of hazelnut cultivars were conducted using the JMP Pro statistical software (version 16.0, USA). Pearson correlation analysis was also performed to examine the relationships among the investigated variables.

## Results and discussions

### Nut and kernel characteristics

The data presented in this study were obtained during the juvenile stage hazelnut cultivars. Consequently, the physical and biochemical disparities identified in this study are indicative of the juvenile stage of cultivar traits under same ecological conditions. Nut and kernel characteristics of hazelnuts include important quality criteria for both fruit breeding and industrial use of hazelnuts [20]. Therefore, determining these characteristics of the varieties and comparing them by growing under similar conditions can be seen as an advantage in variety selection. The procurement of large, aesthetically pleasing nuts is imperative for the in-shell market. Many of the old European cultivars have very large nuts, but a low yield, a long shape, a poor color, and a dull appearance caused by pubescence on the shell [24]. For markets where the aesthetics of a larger kernel are important, such as the in-shell nut market, the smaller size of hazelnuts would be a disadvantage. However, for processing markets, a smaller size could potentially be an advantage, or at least not a disadvantage, and would have minimal impact on processing into oil [25]. Data regarding nut weight, nut width, nut length (NL), nut thickness (NT), and shell thickness during the juvenile stage of the seven varieties examined within the scope of the study are presented in Table 1. The differences between the data obtained in terms of these characteristics in the cultivars were found to be statistically significant (*p* < 0.05). The study revealed that the highest nut weights were observed in the ‘Nocchione’ (3.31 g), ‘Tonda di Giffoni’ (3.27 g), and ‘Tonda Gentile Romana’ (3.22 g) cultivars, respectively, while the difference between these cultivars was found to be insignificant. While there was no significant difference between ‘Çakıldak’ (2.63 g), ‘Mortarella’ (2.54 g), and ‘Foşa’ (2.35 g) cultivars, the difference between the nuts in this group and the other cultivars was significant. The lowest nut weight was observed in the ‘Tombul’ cultivar (1.82 g), and this difference was significant compared to all other cultivars (Table 1).

**Table 1:** Nut characteristics of Türkiye and Italy commercial hazelnut cultivars studied and grown at Ordu (Türkiye)

Cultivars	Nut weight (g)	Nut width (mm)	Nut length (mm)	Nut thickness (mm)	Shell thickness (mm)
‘Mortarella’	2.54 ± 0.14 b	16.88 ± 0.35 de	20.89 ± 0.41 a	13.63 ± 0.44 d	1.35 ± 0.23 bc
‘Nocchione’	3.31 ± 0.74 a	20.57 ± 1.34 b	19.87 ± 1.24 ab	17.27 ± 1.55 ab	1.96 ± 0.23 a
‘Tonda Gentile Romana’	3.22 ± 0.20 a	21.70 ± 0.77 a	18.74 ± 1.10 bc	18.61 ± 1.30 a	1.44 ± 0.07 b
‘Tonda di Giffoni’	3.27 ± 0.16 a	20.94 ± 0.39 ab	19.22 ± 0.28 b	18.30 ± 0.49 a	1.28 ± 0.20 bc
‘Çakıldak’	2.63 ± 0.05 b	18.77 ± 0.16 c	19.20 ± 0.31 b	16.57 ± 0.18 bc	1.24 ± 0.06 bc
‘Foşa’	2.35 ± 0.07 b	17.94 ± 0.43 cd	19.45 ± 0.37 b	15.74 ± 0.25 c	1.19 ± 0.04 bc
‘Tombul’	1.82 ± 0.01 c	16.37 ± 0.32 e	17.77 ± 0.31 c	15.55 ± 0.11 c	1.10 ± 0.09 c

There is no difference between means shown with the same letter in the same column (*p* < 0.05)

In terms of nut dimensional characteristics, nut width was highest in ‘Tonda Gentile Romana’ (21.70 mm) and ‘Tonda di Giffoni’ (20.94 mm) and lowest in ‘Tombul’ (16.37 mm) and ‘Mortarella’ (16.88 mm); nut length was highest in ‘Mortarella’ (20.89 mm) and ‘Nocchione’ (19.87 mm) and lowest in ‘Tombul’ (17.77 mm) and ‘Tonda Gentile Romana’ (18.74 mm); nut thickness was highest in ‘Tonda Gentile Romana’ (18.61 mm), ‘Tonda di Giffoni’ (18.30 mm), ‘Nocchione’ (17.27 mm) and lowest in ‘Mortarella’ (13.63 mm) (Table 1). Shell thickness was found to vary between 1.10 and 1.96 mm. The thinnest shell was found in ‘Tombul’ (1.10 mm), ‘Foşa’ (1.19 mm), and ‘Çakıldak’ (1.24 mm), while ‘Nocchione’ (1.96 mm) was found to have the thickest shell (Table 1).

Data regarding kernel weight, kernel width (Kwi), kernel length (KL), kernel thickness (KT) and kernel percentage during the juvenile stage of the seven varieties examined within the scope of the study are presented in Table 2. The differences between the data obtained in terms of these characteristics in the cultivars were found to be statistically significant (*p* < 0.05). The study revealed that the highest kernel weight was observed in the ‘Tonda di Giffoni’ (1.49 g), ‘Tonda Gentile Romana’ (1.44 g), and ‘Çakıldak’ (1.38 g), respectively, while the difference between these cultivars was found to be insignificant. The lowest kernel weight was observed in the ‘Tombul’ cultivar (0.96 g), and this difference was significant compared to all other cultivars (Table 2).

**Table 2:** Kernel characteristics of Türkiye and Italy commercial hazelnut cultivars studied and grown at Ordu (Türkiye)

Cultivars	Kernel weight (g)	Kernel width (mm)	Kernel length (mm)	Kernel thickness (mm)	Kernel percentage (%)
‘Mortarella’	1.15 ± 0.06 c	12.55 ± 0.22 d	16.43 ± 0.42 a	9.85 ± 0.47 e	45.15 ± 0.88 b
‘Nocchione’	1.28 ± 0.12 bc	14.10 ± 1.02 c	14.61 ± 0.84 bc	11.73 ± 0.80 d	39.61 ± 6.25 c
‘Tonda Gentile Romana’	1.44 ± 0.11 a	16.09 ± 1.23 ab	13.93 ± 0.67 c	14.71 ± 0.44 a	44.78 ± 1.79 b
‘Tonda di Giffoni’	1.49 ± 0.09 a	16.56 ± 0.41 a	14.41 ± 0.47 bc	14.36 ± 0.31 a	45.70 ± 2.20 b
‘Çakıldak’	1.38 ± 0.03 ab	15.31 ± 0.40 b	14.57 ± 0.46 bc	13.54 ± 0.08 b	52.66 ± 0.58 a
‘Foşa’	1.24 ± 0.06 c	14.04 ± 0.50 c	15.29 ± 0.16 b	12.67 ± 0.15 c	52.57 ± 1.57 a
‘Tombul’	0.96 ± 0.02 d	12.77 ± 0.26 d	12.86 ± 0.10 d	12.30 ± 0.33 cd	52.87 ± 1.34 a

There is no difference between means shown with the same letter in the same column (*p* < 0.05)

In terms of kernel dimensional characteristics, kernel width was highest in ‘Tonda di Giffoni’ (16.56 mm), and lowest in ‘Mortarella’ (12.55 mm); kernel length was highest in ‘Mortarella’ (16.43 mm), and lowest in ‘Tombul’ (12.86 mm); nut thickness was highest in ‘Tonda Gentile Romana’ (14.71 mm), and lowest in ‘Mortarella’ (9.85 mm) (Table 2). Kernel percentage was found to vary between 39.61 and 52.87%. The highest kernel percentage was determined in the ‘Tombul’ (52.87%), followed by ‘Foşa’ (52.57%) and ‘Çakıldak’ (52.66%). The lowest kernel percentage was found in ‘Nocchione’ (39.61%) (Table 2).

The following data are presented in Table 3: the number of hazelnuts per 100 g, the number of kernels per 100 g, the weight of 100 hazelnuts and the weight of 100 kernels during the juvenile stage of the seven varieties examined within the scope of the study. The differences between the data obtained in terms of these characteristics in the cultivars were found to be statistically significant (*p* < 0.05). The cultivar ‘Tombul’ (55.16 pcs) exhibited the highest number of nuts in 100 g, while ‘Tonda di Giffoni’ (30.65 pcs) demonstrated the lowest number. Statistical analysis revealed that ‘Tonda di Giffoni’ (30.65 pcs), ‘Tonda Gentile Romana’ (31.12 pcs), and ‘Nocchione’ (31.55 pcs) were positioned within the same group, while ‘Foşa’ (42.62 pcs), ‘Mortarella’ (39.44 pcs) and ‘Çakıldak’ (38.06 pcs) constituted a distinct group (Table 3). Among the cultivars, the highest number of kernels per 100 g was found in ‘Tombul’ (104.40 pcs) and the lowest in ‘Tonda di Giffoni’ (67.16 pcs). For this characteristic, ‘Tonda di Giffoni’ (67.16 pcs), ‘Tonda Gentile Romana’ (69.59 pcs), and ‘Çakıldak’ (72.28 pcs) were in the same group, while ‘Nocchione’ (79.10 pcs), ‘Foşa’ (81.17 pcs), and ‘Mortarella’ (87.36 pcs) formed a separate group (Table 3).

**Table 3:** Number of nuts and kernels in 100 g, and weight of 100 nuts and kernels of Türkiye and Italy commercial hazelnut cultivars studied and grown at Ordu (Türkiye)

Cultivars	Number of nuts in 100 g (pcs)	Number of kernels in 100 g (pcs)	Weight of 100 nuts (g)	Weight of 100 kernels (g)
‘Mortarella’	39.44 ± 2.28 b	87.36 ± 4.84 b	254 ± 14 b	115 ± 6 c
‘Nocchione’	31.55 ± 7.11 c	79.10 ± 8.18 b	331 ± 74 a	128 ± 12 bc
‘Tonda Gentile Romana’	31.12 ± 1.84 c	69.59 ± 5.17 d	322 ± 20 a	144 ± 11 a
‘Tonda di Giffoni’	30.65 ± 1.50 c	67.16 ± 4.09 d	327 ± 16 a	149 ± 9 a
‘Çakıldak’	38.06 ± 0.78 b	72.28 ± 1.67 cd	263 ± 5 b	138 ± 3 ab
‘Foşa’	42.62 ± 1.47 b	81.17 ± 4.40 b	235 ± 7 b	124 ± 6 c
‘Tombul’	55.16 ± 0.29 a	104.40 ± 2.56 a	181 ± 1 c	96 ± 2 d

There is no difference between means shown with the same letter in the same column (*p* < 0.05)

The weight of 100 kernels of the cultivars was determined as follows: ‘Tonda di Giffoni’ (149 g), ‘Tonda Gentile Romana’ (144 g), ‘Çakıldak’ (138 g), ‘Nocchione’ (128 g), ‘Foşa’ (124 g), ‘Mortarella’ (115 g), and ‘Tombul’ (96 g), respectively (Table 3). The highest weight of 100 nuts was found in ‘Nocchione’ and the lowest in ‘Tombul’. Statistically, 3 groups were formed for this characteristic. Accordingly, ‘Nocchione’ (331 g), ‘Tonda di Giffoni’ (327 g), and ‘Tonda Gentile Romana’ (322 g) were in the same group, ‘Çakıldak’ (263 g), ‘Mortarella’ (254 g), and ‘Foşa’ (235 g) were in a separate group and ‘Tombul’ (181 g) with the lowest value formed the third group (Table 3).

When the nut characteristics were analyzed in our study, it was observed that ‘Nocchione’, ‘Tonda di Giffoni’, and ‘Tonda Gentile Romana’, the Italian-origin cultivars, had the heaviest and largest-sized nuts. Turkish cultivars ‘Çakıldak’ and ‘Foşa’ were found to be similar in size and weight to the Italian ‘Mortarella’ cultivar. However, ‘Tombul’ was found to be smaller and lighter than the other cultivars. Again, Italian cultivars ‘Tonda di Giffoni’ and ‘Tonda Gentile Romana’ were found to have the highest kernel weight and kernel size, while similar values were obtained from the ‘Çakıldak’ cultivar. It can also be said that the ‘Tombul’ cultivar has the lowest kernel weight and kernel size. Kernel percentage, a highly heritable trait, shows small variations between trees, years, and locations and is therefore considered an important trait for variety identification [24]. Kernel percentage is affected by shell thickness and kernel filling of the nut. In terms of this trait, all Turkish cultivars were found to be higher than Italian cultivars.

In a previous study conducted under Viterbo (Italy) conditions, nut weight, kernel weight, and kernel percentage were reported as 3.11 g, 1.22 g, and 39.59% for ‘Nocchione’; 2.88 g, 1.35 g, and 46.39% for ‘Tonda Gentile Romana’; 3.22 g, 1.39 g, and 43.11% for ‘Tonda di Giffoni’; and 1.48 g, 0.79 g, and 49.55% for ‘Tombul’ [26]. In another study carried out under Italian conditions, the nut weight of the ‘Mortarella’ cultivar was 2.42–2.71 g, and the kernel weight was 1.07–1.24 g [27]. In the study conducted in the Eastern Black Sea region (Türkiye), nut weight, kernel weight, kernel weight, kernel percentage, and shell thickness were reported respectively as 1.81 g, 0.98 g, 54.2%, 0.83 mm for ‘Tombul’, 1.90 g, 0.98 g, 51.5%, 1.05 mm for ‘Çakıldak’, and 1.91 g, 1.03 g, 53.6%, 1.03 mm for ‘Foşa’ [28]. In this context, the results obtained in terms of shell and kernel fruit physical properties were generally consistent with the literature. In this study, significant differences were found between hazelnut cultivars originating from Türkiye and Italy, grown under the same ecological conditions. These differences are important, especially in terms of industrial use of hazelnuts, consumer demands, and establishment of breeding programs. It can be said that Italian hazelnut varieties such as ‘Nocchione’, ‘Tonda di Giffoni’, and ‘Tonda Gentile Romana’ stand out with their high fruit weights and fruit sizes. This indicates that they are highly attractive hazelnuts, especially for in-shell market consumption. For all that Turkish cultivars ‘Çakıldak’ and ‘Foşa’ were close to Italian cultivars in terms of fruit size and weight. However, the high shell thickness of Italian varieties causes a lower kernel percentage. In this respect, all Turkish hazelnut cultivars stood out. Although they generally have low fruit weight, their thinner shells and high kernel percentage provide an extra advantage for the processing industry.

### Protein, oil, and ash contents

Hazelnuts play an important role in human nutrition and health due to their unique nutritional value. Hazelnuts provide 600–650 kcal per 100 g, mainly due to fat (43–73%), protein (10–25%), and carbohydrate (10–20%) content [29]. It also contains organic acids, cellulose, pectin, vitamins, and unsaturated fatty acids. Hazelnut is also a rich source of several bioactive compounds such as L-arginine, selenium, caffeic acid, gallic acid, p-hydroxybenzoic acid, epicatechin, sinapic acid, and quercetin, which may have anti-atherogenic effects through biological mechanisms acting on different pathways of cardiovascular disease development [30]. The data pertaining to the crude protein content, crude fat content and ash content of the seven varieties examined during the juvenile stage of the study are presented in Table 4. The differences between the data obtained in terms of these characteristics in the cultivars were found to be statistically significant (*p* < 0.05). Accordingly, the protein content was found to be lower in ‘Mortarella’ (15.53%), ‘Foşa’ (15.75%), and ‘Tonda di Giffoni’ (15.88%), while it was higher in ‘Nocchione’ (18.78%), ‘Çakıldak’ (18.40%), and ‘Tonda Gentile Romana’ (17.95%). The oil content was lower in ‘Nocchione’ (52.46%), ‘Tonda Gentile Romana’ (53.44%), ‘Mortarella’ (55.23%), and ‘Tonda di Giffoni’ (55.90%), while it was higher in ‘Tombul’ (65.74%), ‘Foşa’ (62.29%), and ‘Çakıldak’ (59.80%). Ash content was lower in ‘Mortarella’ (2.18%), ‘Tonda di Giffoni’ (2.21%), ‘Nocchione’ (2.22%), and ‘Tonda Gentile Romana’ (2.24%) and higher in ‘Foşa’ (2.51%), ‘Tombul’ (2.46%), and ‘Çakıldak’ (2.36%) (Table 4). Previously, the protein content of Palaz, ‘Tombul’, Kara, and ‘Çakıldak’ hazelnut cultivars was reported as 18.25–22.06%, fat content as 57.39–62.90%, and ash content as 2.22–2.36% [31]; fat content of ‘Tonda Gentile Romana’, ‘Tonda di Giffoni’, and ‘Nocchione’ cultivars was reported as 48.38–49.65% [32]; protein content of 12 different hazelnut cultivars as 14.66–24.61%, fat content as 43.22–68.44% and ash as 2.62–4.13% [33]. The findings obtained from the study were consistent with the literature. The differences observed between cultivars in terms of these traits provide important information to breeders and industry stakeholders about processing techniques, oil production, and cultivar selection for increasing nutritional values.

**Table 4:** Protein, oil, and Ash contents of Türkiye and Italy commercial hazelnut cultivars studied and grown at Ordu (Türkiye)

Cultivars	Protein (%)	Oil content (%)	Ash (%)
‘Mortarella’	15.53 ± 0.74 d	55.23 ± 3.62 cd	2.18 ± 0.09 d
‘Nocchione’	18.78 ± 0.62 a	52.46 ± 3.70 d	2.22 ± 0.04 d
‘Tonda Gentile Romana’	17.95 ± 0.45 bc	53.44 ± 3.73 cd	2.24 ± 0.03 cd
‘Tonda di Giffoni’	15.88 ± 0.70 d	55.90 ± 4.37 c	2.21 ± 0.06 d
‘Çakıldak’	18.40 ± 0.76 ab	59.80 ± 1.40 b	2.36 ± 0.13 bc
‘Foşa’	15.75 ± 0.39 d	62.29 ± 1.12 b	2.51 ± 0.11 a
‘Tombul’	17.33 ± 0.36 c	65.74 ± 2.00 a	2.46 ± 0.23 ab

There is no difference between means shown with the same letter in the same column (*p* < 0.05)

### Biochemical characteristics

Phenolic compounds play an important role in reducing the risk of human disease. The antioxidant properties of phenolic compounds have been demonstrated to be effective against a range of pathological problems associated with oxidative stress damage. Furthermore, bioactive compounds in plants have been shown to possess anti-inflammatory, antiulcer, antiallergic, antimicrobial, antithrombotic, antiatherogenic, and anticarcinogenic properties [12]. Total phenolics (g GAE kg^− 1^), total flavonoids (g QE kg^− 1^), DPPH (mmol TE kg^− 1^) and FRAP (mmol TE kg^− 1^) data obtained during the juvenile stage of the seven cultivars examined in the study are presented in Table 5. The differences between the data obtained in terms of these characteristics in the cultivars were found to be statistically significant (*p* < 0.05). Accordingly, the TPC was found to be higher in ‘Tombul’ (50.86 g GAE kg^− 1^), ‘Foşa’ (49.49 g GAE kg^− 1^), and ‘Çakıldak’ (48.61 g GAE kg^− 1^), while the lowest was recorded in ‘Tonda di Giffoni’ (21.84 g GAE kg^− 1^). The TFC was found to be higher in ‘Tombul’ (6.09 g QE kg^− 1^) and ‘Tonda Gentile Romana’ (6.07 g QE kg^− 1^), while it was lower in ‘Nocchione’ (3.11 g QE kg^− 1^) and ‘Tonda di Giffoni’ (3.24 g QE kg^− 1^) (Table 5). Total phenolic content was previously reported as 4.32 g GAE kg^− 1^ in ‘Tombul’, 1.78 g GAE kg^− 1^ in ‘Foşa’, and 2.46 g GAE kg^− 1^ in ‘Çakıldak’ [34]; 1.25 g GAE kg^− 1^ in ‘Nocchione’, 1.42 g GAE kg^− 1^ in ‘Tonda di Giffoni’, and 1.56 g GAE kg^− 1^ in ‘Tonda Gentile Romana’ [35]. The Turkish cultivars analyzed in this study were found to be richer than all Italian cultivars in total phenolics. ‘Tombul’, ‘Foşa’, and ‘Çakıldak’ cultivars had the highest values, respectively. In the study, ‘Tombul’ and ‘Tonda Gentile Romana’ cultivars were found to have the highest values in terms of total flavonoid content. Balik [36] determined the total flavonoid content in Turkish hazelnut cultivars between 0.07 and 0.65 g GAE kg^− 1^ and reported 0.34 g GAE kg^− 1^ in ‘Tombul’, 0.12 g GAE kg^− 1^ in ‘Çakıldak’ and 0.73 g GAE kg^− 1^ in ‘Foşa’. The antioxidant activity was highest in ‘Çakıldak’ (37.51 mmol TE kg^− 1^) and lowest in ‘Tonda di Giffoni’ (13.15 mmol TE kg^− 1^) according to the DPPH assay, and highest in ‘Çakıldak’ (102.13 mmol TE kg^− 1^) and lowest in ‘Tombul’ (57.22 mmol TE kg^− 1^) according to the FRAP assay (Table 5). In this study, the antioxidant activity was determined according to DPPH and FRAP assays. The results of both tests showed that antioxidant activity was lower in ‘Tonda di Giffoni’ and ‘Tombul’ cultivars, while the ‘Çakıldak’ cultivar had the highest antioxidant activity. Balik [36] determined the antioxidant activity according to DPPH and FRAP assays, respectively, for ‘Tombul’ 2.46 mmol TE kg^− 1^ and 12.18 mmol TE kg^− 1^, for ‘Çakıldak’ 2.53 mmol TE kg^− 1^ and 19.50 mmol TE kg^− 1^, and ‘Foşa’ 1.73 mmol TE kg^− 1^ and 2.97 mmol TE kg^− 1^; Balta et al. [37]reported between 39.05 and 70.27 mmol TE kg^− 1^ according to FRAP and between 25.54 and 37.37 mmol TE kg^− 1^ according to DPPH in ‘Çakıldak’. The results obtained were compatible with the values reported by Balta et al. [37] but significantly higher than the values reported by Balik [36]. This difference may be due to geographical origin [38], cultural practices, ecological conditions, and methodological differences in the determination of antioxidant activity [11].

**Table 5:** Total phenolics, total flavonoids, and antioxidant activity (DPPH and FRAP assays) of Türkiye and Italy commercial hazelnut cultivars studied and grown at Ordu (Türkiye)

Cultivars	Total phenolics (g GAE kg^− 1^)	Total flavonoids (g QE kg^− 1^)	DPPH (mmol TE kg^− 1^)	FRAP (mmol TE kg^− 1^)
‘Mortarella’	40.00 ± 20.78 ab	4.41 ± 0.30 b	19.01 ± 10.46 bc	70.71 ± 41.40 ab
‘Nocchione’	36.61 ± 6.13 ab	3.11 ± 0.39 c	29.54 ± 18.20 a-c	79.78 ± 16.70 ab
‘Tonda Gentile Romana’	41.10 ± 3.18 ab	6.07 ± 1.21 a	34.01 ± 25.65 ab	76.54 ± 17.18 ab
‘Tonda di Giffoni’	21.84 ± 8.53 b	3.24 ± 0.71 c	13.15 ± 1.02 c	57.77 ± 1.71 b
‘Çakıldak’	48.61 ± 32.40 a	4.94 ± 1.36 b	37.51 ± 27.23 a	102.13 ± 68.07 a
‘Foşa’	49.49 ± 19.38 a	4.00 ± 0.78 bc	17.23 ± 6.67 bc	69.21 ± 24.17 ab
‘Tombul’	50.86 ± 1.71 a	6.09 ± 0.54 a	14.51 ± 5.06 c	57.22 ± 7.88 b

There is no difference between means shown with the same letter in the same column (*p* < 0.05)

### Fatty acid composition

Palmitic acid (C_16:0_) (PA), stearic acid (C_18:0_) (SA), oleic acid (C_18:1_) (OA), and linoleic acid (C_18:2_) (LA) data obtained during the juvenile stage of the seven cultivars examined in the study are presented in Table 6. The differences between the data obtained in terms of these characteristics in the cultivars were found to be statistically significant (*p* < 0.05). Accordingly, the cultivars with high palmitic acid content were ‘Çakıldak’ (5.79%), ‘Tonda Gentile Romana’ (5.78%), ‘Tombul’ (5.49%), and ‘Foşa’ (5.16%), respectively, and the lowest was ‘Tonda di Giffoni’ (4.66%); the cultivars with high stearic acid content were ‘Çakıldak’ (2.76%), ‘Foşa’ (2.75%), ‘Tombul’ (2.65%), and ‘Tonda Gentile Romana’ (2.63%), respectively, and the lowest was ‘Nocchione’ (1.71%); the cultivars with high oleic acid content were ‘Nocchione’ (87.37%), ‘Tonda Gentile Romana’ (87.01%), ‘Tonda di Giffoni’ (86.77%), and ‘Çakıldak’ (86.24%), the lowest was ‘Tombul’ (83.21%); the cultivars with high linoleic acid content were ‘Tombul’ (8.77%), ‘Tonda di Giffoni’ (5.70%), ‘Mortarella’ (5.59%), and ‘Foşa’ (5.09%), the lowest was ‘Tonda Gentile Romana’ (4.25%). In related studies, the fatty acid composition of hazelnut cultivars, Cristofori et al. [32] was reported palmitic acid 7.06%, stearic acid 2.55%, oleic acid 79.78%, and linoleic acid 10.52% in ‘Tonda Gentile Romana’, palmitic acid 6.34%, stearic acid 3.32%, oleic acid 80.85%, and linoleic acid 9.96% in ‘Tonda di Giffoni’; palmitic acid 5.29%, stearic acid 2.22%, oleic acid 83.66%, and linoleic acid 7.48% in ‘Nocchione’; Balik [36] was reported palmitic acid 7.46%, stearic acid 4.89%, oleic acid 80.03%, and linoleic acid 7.40% in ‘Çakıldak’; palmitic acid 7.71%, stearic acid 4.06%, oleic acid 78.19%, and linoleic acid 9.83% in ‘Tombul’; palmitic acid 6.69%, stearic acid 4.48%, oleic acid 80.70%, and linoleic acid 7.98% in ‘Foşa’; Yaman et al. [39] was reported palmitic acid 6.09%, stearic acid 2.41%, oleic acid 77.10%, and linoleic acid 13.33% in ‘Çakıldak’; palmitic acid 5.73%, stearic acid 2.17%, oleic acid 71.18%, and linoleic acid 19.03% in ‘Tombul’. Accordingly, the results obtained from this study were consistent with the literature. Fatty acid composition is affected by many factors such as genetic structure, ecological condition, geographical location, cultural practices, harvest time, storage methods, drying techniques, and maturity stages [12]. Previously, fatty acid profiles showed differences in both Italian and Turkish hazelnut cultivars, and similar results were observed in our study. This may be due to the genetic structure of the cultivars.

**Table 6:** Fatty acid composition of Türkiye and Italy commercial hazelnut cultivars studied and grown at Ordu (Türkiye)

Cultivars	Palmitic acid (C_16:0_) (%)	Stearic acid (C_18:0_) (%)	Oleic acid (C_18:1_) (%)	Linoleic acid (C_18:2_) (%)
‘Mortarella’	4.85 ± 0.16 d	2.39 ± 0.06 d	85.94 ± 1.02 a	5.59 ± 0.22 b
‘Nocchione’	4.67 ± 0.11 e	1.71 ± 0.07 e	87.37 ± 1.28 a	4.27 ± 0.15 d
‘Tonda Gentile Romana’	5.78 ± 0.12 a	2.63 ± 0.09 c	87.01 ± 1.66 a	4.25 ± 0.15 d
‘Tonda di Giffoni’	4.66 ± 0.12 e	2.30 ± 0.08 d	86.77 ± 1.06 a	5.70 ± 0.24 b
‘Çakıldak’	5.79 ± 0.10 a	2.76 ± 0.15 a	86.24 ± 1.51 a	4.80 ± 0.25 c
‘Foşa’	5.16 ± 0.09 c	2.75 ± 0.13 ab	86.03 ± 0.93 a	5.09 ± 0.38 c
‘Tombul’	5.49 ± 0.21 b	2.65 ± 0.08 bc	83.21 ± 2.80 b	8.77 ± 0.14 a

There is no difference between means shown with the same letter in the same column (*p* < 0.05)

### Principal components and correlation analysis

**Fig. 2:**
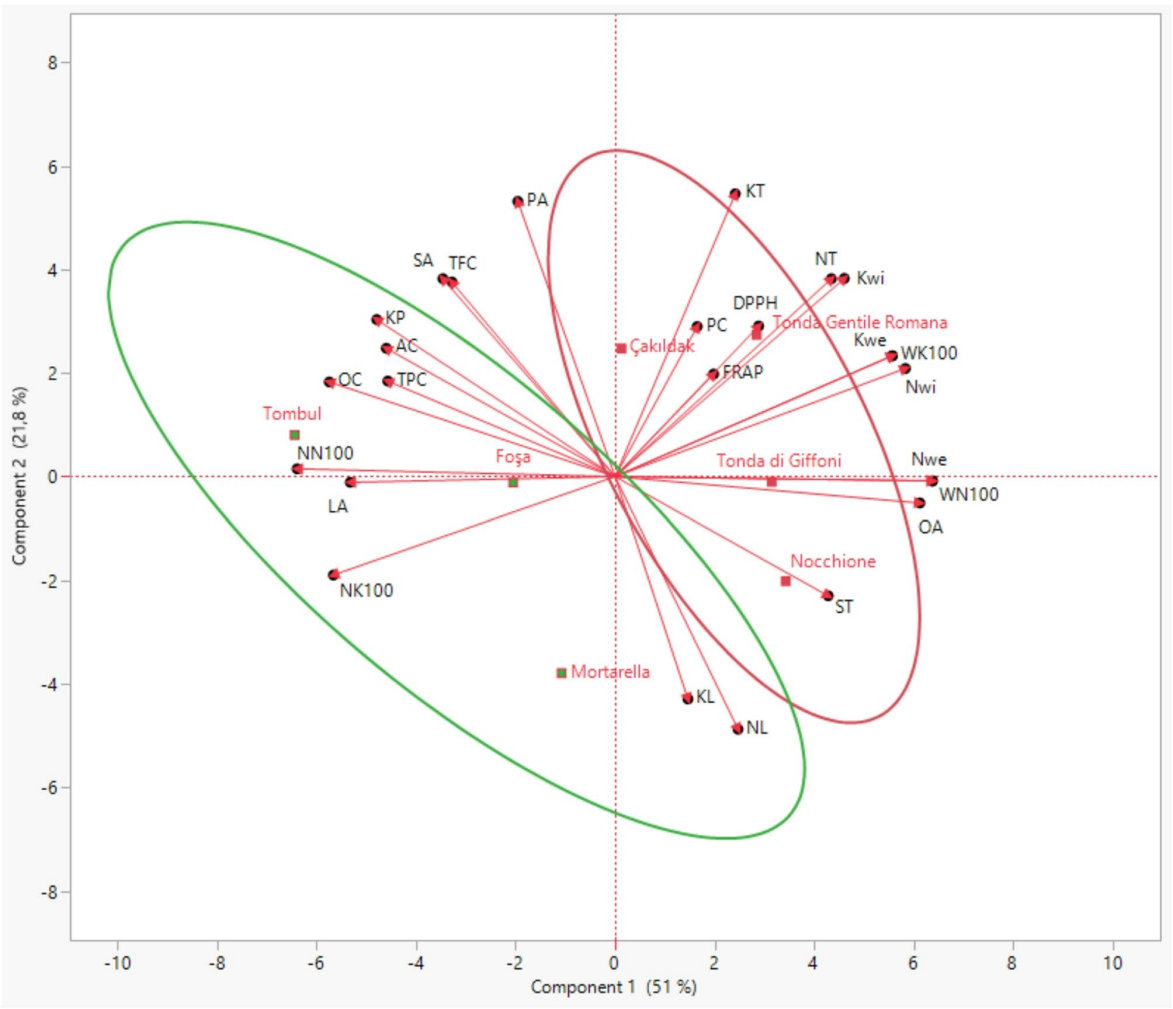
Biplot graph of the first two principal components in Türkiye and Italy commercial hazelnut cultivars investigated

The twenty-five characteristics were used for principal components analysis (PCA), and 4 PCs had eigenvalues higher than 1.0. These components explained 94.6% of the total variation. PC1, which explained 51.0% of the total variability, was mainly related to WK100, Kwe, Kwi, NK100, Nwi, NT, KT, NN100, WN100, Nwe, OA, LA, and TPC. The most important characteristics that affected PC1 were WK100 and Kwe (0.98). Explaining 21.8% of the variability, PC2 was related to WN100, Nwe, KP, ST, SA, OC, and AC. KP had the most effect on PC2 (0.96). PC3 was related to KL and NL by explaining 12.1% of the variability. PC4, which is effective on PC and antioxidant properties, including FRAP and DPPH assays, explained 9.7% of the total variability. According to the PCA results, Tombul, Foşa, ve Mortarella cultivars were grouped in terms of KP, TPC, TFC, OC, AC, NN100, NK100, LA, KL, and NL, Çakıldak, Tonda di Giffoni, Nocchione, Tonda Gentile Romana cultivars were grouped by antioxidant activity, physical traits, PC, ST, and OA (Fig. 2; Table 7). Similarly, Karakaya [12] reported that the first two components formed by PCA analysis explained 61.6% of the total variation in ‘Tombul’ cultivars. The study also reported a strong association between physical characteristics. As in our study, physical characteristics make a strong contribution to PC1, while biochemical characteristics contribute to subsequent components.

**Table 7:** The principal component of Türkiye and Italy commercial hazelnut cultivars investigated

Traits	PC1	PC2	PC3	PC4
WK100	**0.975673**	−0.118320	0.091846	0.130846
Kwe	**0.975673**	−0.118320	0.091846	0.130846
Kwi	**0.967573**	0.009248	−0.230881	0.047562
NK100	**−0.952087**	0.125567	−0.199226	−0.178669
Nwi	**0.841073**	−0.474939	−0.211414	0.098764
NT	**0.796903**	−0.228982	−0.546408	0.059656
KT	**0.796342**	0.241681	−0.527847	0.020562
NN100	**−0.789529**	0.534707	−0.248211	−0.131579
WN100	**0.746892**	**−0.640688**	0.070851	0.090995
Nwe	**0.746075**	**−0.642235**	0.066937	0.090073
OA	**0.723701**	−0.474765	0.370357	0.233790
LA	**−0.637125**	0.331827	−0.454350	−0.432052
TPC	**−0.558391**	0.510220	−0.049120	0.451591
KP	−0.210053	**0.962238**	−0.132655	0.033564
ST	0.091686	**−0.856250**	0.035381	0.341871
SA	0.036668	**0.849161**	0.016087	−0.000338
OC	−0.471884	**0.814293**	−0.308709	−0.120279
AC	−0.311497	**0.774028**	−0.281200	0.040673
KL	0.004372	−0.098149	**0.984697**	−0.019935
NL	−0.004707	−0.361787	**0.901215**	0.048025
FRAP	0.210963	0.078536	0.232327	**0.944780**
DPPH	0.310868	−0.176341	−0.000512	**0.898271**
PC	0.046723	−0.301829	**-**0.525841	0.792328
TFC	−0.248286	0.276963	−0.363567	0.161764
PA	0.049702	0.478593	−0.306595	0.495098
Eigenvalue	12.7	5.5	3.0	2.4
Variance	51.0	21.8	12.1	9.7
Cumulative variance	51.0	72.8	84.9	94.6

Factor loading ≥|0.55| are marked in bold

**Fig. 3:**
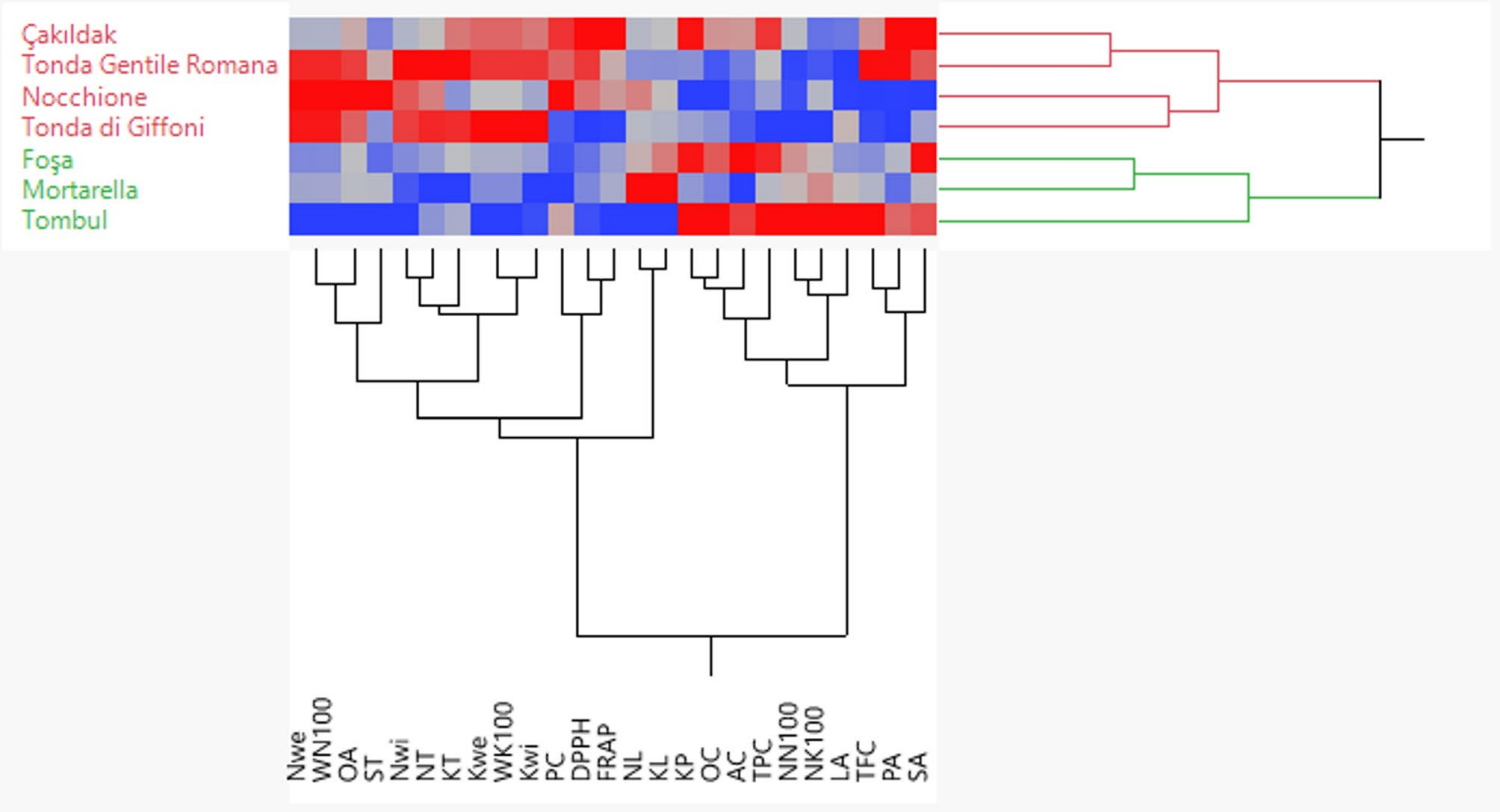
Heatmap analysis and grouping of Türkiye and Italy commercial hazelnut cultivars investigated based on nut traits, bioactive compounds, and fatty acid composition

PCA results are largely consistent with hierarchical heatmap clustering (Fig. 3). Accordingly, the hazelnut cultivars investigated were divided into two groups (A and B). The first group (A) consisted of ‘Çakıldak’, ‘Tonda Gentile Romana’, ‘Nocchione’, and ‘Tonda di Giffoni’. The cultivars in this group were generally characterized by higher values of fruit physical properties, oleic acid, protein, and antioxidant activity. In this context, it can be said that the cultivars in this group have a very suitable profile for table consumption. The second group (B) consisted of ‘Tombul’, ‘Foşa’, and ‘Nocchione’. The cultivars in group B were characterized by higher levels of biochemical compounds. Within this group, the ‘Tombul’ cultivar has a unique biochemical content compared to all other cultivars, with high mean values for kernel percentage, oil content, ash content, total phenolic content, linoleic acid, and total flavonoid content (Fig. 3). Accordingly, it can be said that the cultivars in this group are suitable for industrial use in terms of their physical properties and can be good sources for enrichment of nutrient contents in terms of breeding programs, especially ‘Tombul’ cultivar.

**Fig. 4:**
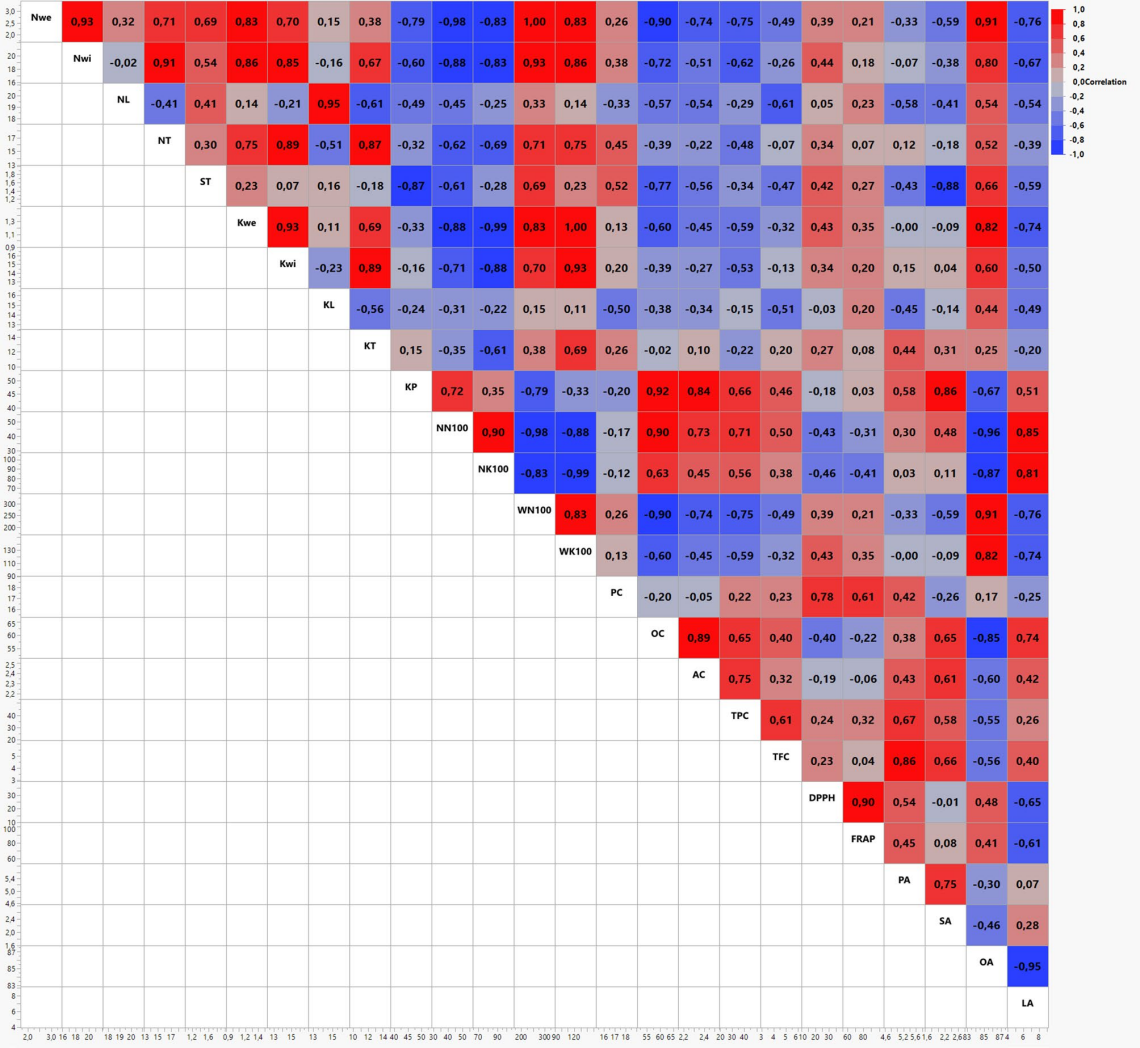
Correlation among the examined characteristics of Türkiye and Italy commercial hazelnut cultivars investigated

Correlations between the characteristics investigated are presented in Fig. 4. The nut weight had a very strong positive correlation with nut width (*r* = 0.93**) and oleic acid (*r* = 0.91**), and had a strong correlation with kernel weight (*r* = 0.83*). In contrast, it had a very strong negative correlation with oil content (*r* = − 0.90**) and a strong negative correlation with kernel percentage (*r* = − 0.79**) and linoleic acid (*r* = − 0.76*). The kernel percentage had a very strong positive correlation with oil content (*r* = 0.92**), a strong positive correlation with stearic acid (*r* = 0.86*), and ash content (*r* = 0.84*). The protein content had a strong positive correlation with DPPH (*r* = 0.78*). The oil content had a strong positive correlation with ash content (*r* = 0.89**), but a strong negative correlation with oleic acid (*r* = − 0.85*). TFC had a strong positive correlation with palmitic acid (*r* = 0.86*), DPPH had a very strong positive correlation with FRAP (*r* = 0.90**), and oleic acid had a very strong negative correlation with linoleic acid (*r* = −0.95**). In related studies, positive correlations have been reported between DPPH and FRAP [11], and between nut weight, kernel weight, nut and kernel dimensions in hazelnuts [40]. Similarly to our study, a strong negative correlation between oleic and linoleic acids in hazelnuts has been reported by Yaman et al. [39]. Furthermore, it has been reported that oil content has a strong negative correlation with linoleic acid content, while there is a positive correlation between dry matter content and antioxidant activity [41].

## Conclusions

This study reveals that Turkish and Italian hazelnut cultivars grown under the same ecological conditions show significant differences in physical and biochemical characteristics during the juvenile stage. In the study, Italian cultivars were found to have larger fruits and higher kernel weight. This indicates that cultivars have an advantage in terms of the in-shell market for fresh consumption. Turkish cultivars, on the other hand, were characterized by relatively smaller fruits, but higher kernel percentage and richer biochemical contents. This shows that these cultivars are more advantageous for industrial use. The study findings contribute to the determination of which cultivar can be used for oil processing, and which variety can be used as kernel for nutritional content, depending on the ecology in which it is grown. It can be said that the results of this comparative analysis provide important information, especially for breeding programs and cultivar selection.

## Data Availability

Data will be available on request to the corresponding authors.

## Abbreviations

AA: Antioxidant activity
AC: Ash content
DPPH: 1,1-diphenyl-2-picrylhydrazyl
FAMEs: Fatty acid methyl esters
FID: Flame ionization detector
FRAP: Ferric reducing antioxidant power
g: Gram
GAE: Gallic acid equivalent
GC: Gas chromatography
ha: hectares
kcal: Kilocalorie
KL: Kernel length
KP: Kernel percentage
KT: Kernel thickness
Kwe: Kernel weight
Kwi: Kernel width
LA: Linoleic acid (C_18_:_2_)
m: Meter
mm: Millimeters
N: Nitrogen
NK100: Number of kernels in 100 g
NL: Nut length
NN100: Number of nuts in 100 g
NT: Nut thickness
Nwe: Nut weight
Nwi: Nut width
OA: Oleic acid (C_18_:_1_)
OC: Oil content
PA: Palmitic acid (C_16_:_0_)
PC: Protein content
PCA: Principal components analysis
pcs: Pieces
SA: Stearic acid (C_18_:_0_)
ST: Shell thickness
t: Ton
TE: Trolox equivalent
TFC: Total flavonoid content
TPC: Total phenolic content
WK100: Weight of 100 kernels
WN100: Weight of 100 nuts
